# Diversity and Species Richness of Lepidopteran Stemborers and Their Host Plants Across Major Vegetation Mosaics of East Africa

**DOI:** 10.1002/ece3.74314

**Published:** 2026-09-13

**Authors:** Elie Ntirenganya, Muluken Goftishu, Yoseph Assefa, Bonoukpoè Mawuko Sokame, Simon Boniface Boni, Janvier Uwayezu, Hategekimana Athanase, Venuste Nsengimana

**Affiliations:** ^1^ Africa Center of Excellence for Climate‐Smart Agriculture and Biodiversity Conservation Haramaya University, Haramaya Dire Dawa Dire Dawa Ethiopia; ^2^ Center of Excellence in Biodiversity and Natural Resource Management (CoEB) University of Rwanda Kigali Rwanda; ^3^ Department of Crop Production, Faculty of Agriculture University of Eswatini Kwaluseni Eswatini; ^4^ International Centre of Insects Physiology and Ecology (ICIPE) Nairobi Kenya; ^5^ The World Vegetable Center Eastern and Southern Africa Arusha Tanzania; ^6^ Ecology and Evolutionary Biology Department University of California Santa Cruz California USA; ^7^ Rwanda Agriculture and Animal Resources Development Board (RAB) Kigali Rwanda

**Keywords:** ecological mosaics, Lepidoptera, network, stem feeders, wild habitat

## Abstract

The study was carried out in the major ecological vegetation mosaics of Rwanda, including Afro‐alpine mountain mosaics within the Guineo–Congolese region in the north; Akagera sub‐humid savanna mosaics in the east; montane forest mosaics in the south; and modified urban wetland and riparian mosaics of Kigali City, within the Sudanian–Guinea–Congo transition zone. At each selected locality, purposive sampling in (i) edges of the cultivated field and adjacent uncultivated lands, (ii) open patches along the forest access roads and pathways, (iii) riparian zones along urban areas, rivers, and streams, (iv) edge of lakes, and (v) swamp ecosystems was conducted to detect stemborer infestations. A total of 6098 larvae belonging to 18 genera representing five families were recovered from 56 wild host plant species. The abundance of stemborers and host plants was higher in modified urban wetlands and its riparian zones (41.7%, *N* = 2536; 47 host plants) and lower in Afro‐alpine mountain mosaics (10.3% of larvae; seven host plants). The Poaceae family hosted 62.5% of all stemborers, with *Pennisetum purpureum* being the most affected host, accounting for 29.53% (*N* = 1801). Two major economically important pests in sub‐Saharan Africa dominated collections: *Busseola fusca* (47.11%, *N* = 2873) and *Chilo partellus* (17.61%, *N* = 1074). Additionally, five new stemborer‐host plant associations and a fruit‐feeder genus of *Thaumatotibia* were observed associated with stems. These results are preliminary indicators of possible new stemborer species and host use that warrant further investigation. Our findings provide a baseline assessment of lepidopteran stemborer diversity and host plant associations across regional vegetation mosaics, highlighting the importance of natural and semi‐natural habitats in supporting stemborer communities. The study also reveals previously undocumented plant associations that warrant further ecological and taxonomic investigation.

## Graphical abstract

Lepidopteran stemborer populations in wild habitats are increasingly influenced by anthropogenic disturbances, including urbanization and agricultural intensification in addition to climate change, which alter community composition and diversity within fragmented native ecosystems. A survey across Rwandan vegetation mosaics revealed that modified Kigali urban wetlands and riparian habitats harbor the highest stemborer diversity and abundance, with economically important species such as *Busseola fusca* and *Chilo partellus* predominating. The study further observed novel host plant associations and evidence of expanded host utilization, suspecting more host expansion than previously recognized among key stemborer species.

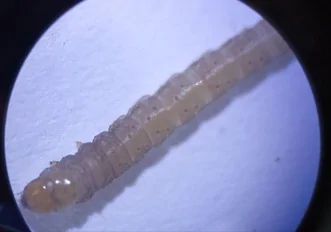

## 1 Introduction

Current estimations indicate that more than 150 species of lepidopteran stemborer have been described globally, and are differently distributed across regions (Kfir et al. 2002; Sallam 2006; Moolman et al. 2014; Borges Filho et al. 2019). East African surveys encompassing Eritrea, Ethiopia, Kenya, Madagascar, Mozambique, Tanzania, Uganda, and Zanzibar reported more than 135 species (Le Rü et al. 2006b). Other surveys conducted in Ethiopia reported 44 species (Goftishu et al. 2018), in the Democratic Republic of Congo reported 20 species (Kankonda et al. 2017), in South Africa and Mozambique reported 50 and 39 species (Moolman et al. 2014). West Africa is less represented, where 32 species were recorded in Cameroon (Ong'amo et al. 2014). These species were primarily in three dominant lepidopteran families, namely Noctuidae, Crambidae, and Pyralidae (Le Rü et al. 2006a, 2006b; Moyal 2014), while few representatives from Tortricidae (Moyal 2014) and Thyrididae families (Moolman et al. 2014) were reported.

Stemborers exhibit a broad host range that extends beyond cultivated cereals to numerous wild vegetation, which serve as alternative hosts (Overholt and Goebel 2001). Stemborers host range surveys across East Africa reported over 75 wild host plants (Le Rü et al. 2006a); in Southern Africa, reported over 65 (Moolman et al. 2014), while only 22 wild host plant species were listed in West Africa, particularly in Cameroon (Ndemah et al. 2007; Ong'amo et al. 2014; Oben et al. 2015). Most of these reported wild host plants were from Poaceae, Cyperaceae, and Typhaceae families (Le Rü et al. 2006a, 2006b; Assefa et al. 2010; Moolman et al. 2014; Goftishu et al. 2018), where Poaceae is the dominant family, particularly *Pennisetum*, *Panicum*, *Sorghum*, and *Phragmites* genera (Haile and Hofsvang 2002; Goftishu et al. 2018; Moeng et al. 2018). Few genera of the Cyperaceae and Typhaceae families were reported as well (Ndemah et al. 2007; Ong'amo et al. 2014; Oben et al. 2015). Other studies reported that stemborer populations can move from wild to cultivated plants due to variation in ecological vegetation mosaics and seasonal conditions (Maicher et al. 2018; Morshed et al. 2023; Ntirenganya et al. 2026).

Vegetation mosaics in East Africa play a decisive role in the structure of stemborer communities due to differences in vegetation types, habitat heterogeneity, and land use patterns which design and shape stemborer community structure and host interactions (Ong'amo et al. 2014; Goftishu et al. 2018). Recent surveys classified stemborers as generalist oligophagous species such as *Busseola fusca* and *Chilo partellus* that colonize or associated with multiple vegetation mosaics (Goftishu et al. 2018; Ong’amo et al. 2018). This reflects its adaptability to various crops, grasses, fodder, and wetland vegetation. In addition, *Sesamia* species remain largely confined to humid or semi‐aquatic habitats (Overholt and Goebel 2001), while sugarcane growing zones are dominated by *Eldana saccharina* (Assefa 2006; Assefa et al. 2010; Ntirenganya et al. 2025). However, recent changes in land use and vegetation fragmentation activities have pushed a range of stemborer expansions and habitat shifts to new ecological zones and host plants. These trends are reported in *B. fusca* and *C. partellus* in Ethiopia, for example (Getu et al. 2001; Tamiru et al. 2007; Asmare et al. 2014); Kenya (Kanya et al. 2004; Ong'amo 2005, 2009; Otieno et al. 2008); and in other regions (Le Rü et al. 2006b; Tamiru et al. 2007; Kankonda et al. 2017; Ntirenganya et al. 2026).

Considering the ecological regions and vegetation mosaics of Rwanda, Rwanda's natural vegetation is a regional mosaic comprising Guineo–Congolese in the western and northern parts, Sudanian forests in the eastern parts, and Guineo–Congolese–Sudanian transition zones in the central parts and Kigali City (White 1983; SANBI et al. 2022; Lillesø et al. 2024). These ecological zones are marked with different vegetation and habitat heterogeneity, where Sudanian forests are characterized by a mixture of shrub‐savanna and grassland ecosystems dominated by tall perennial grasses comprising *Andropogon* spp., *Hyparrhenia* spp., and *Brachiaria* spp. (Lillesø et al. 2024; SANBI et al. 2022). These grasses form dense herbaceous layers supporting grazing animals (MINAGRI 2021; SANBI et al. 2022). In contrast, Guineo‐Congolian vegetation mosaics feature a blend of semi‐evergreen forests and open grasslands dominated by *Hyparrhenia ssp*., *Imperata* ssp., and *Pennisetum* spp. (MINAGRI 2021; SANBI et al. 2022; White 1983). The Guineo–Congolese–Sudan transition zones are marked by intersection of mixed vegetation of grasslands, bushlands, forests, and rich water bodies (Lillesø et al. 2024; Morpurgo et al. 2024).

Despite this rich ecological diversity and heterogeneous vegetation mosaics, knowledge of lepidopteran stemborers in Rwanda remains limited. Recent studies have reported the occurrence of *E. saccharina* in sugarcane plantations (Ntirenganya et al. 2025), while a taxonomic investigation described *S. kabirara* Le Rü as a new species associated with grasses in the wetlands of Kigali City and as reported in similar habitats of neighboring countries (Hévin et al. 2024). Furthermore, regional surveys have documented *Poeonoma* spp. as common stemborers associated with *P. purpureum* (Poaceae: Panicoideae: Paniceae) in the western part of Rwanda (Le Rü et al. 2020). However, these studies were geographically restricted or taxonomically focused and did not examine how ecological variation, vegetation composition, and habitat heterogeneity shape stemborer community structure and host plant associations across Rwanda. To address these knowledge gaps, the present study investigates the diversity of lepidopteran stemborers and their associated wild host plants across different vegetation mosaics that represent major ecological regions of Rwanda. In addition, we explore stemborer–host plant interaction networks to provide a preliminary assessment of community structure and to discuss the potential implications of habitat modification and fragmentation on stemborer community structure.

**FIGURE 1:**
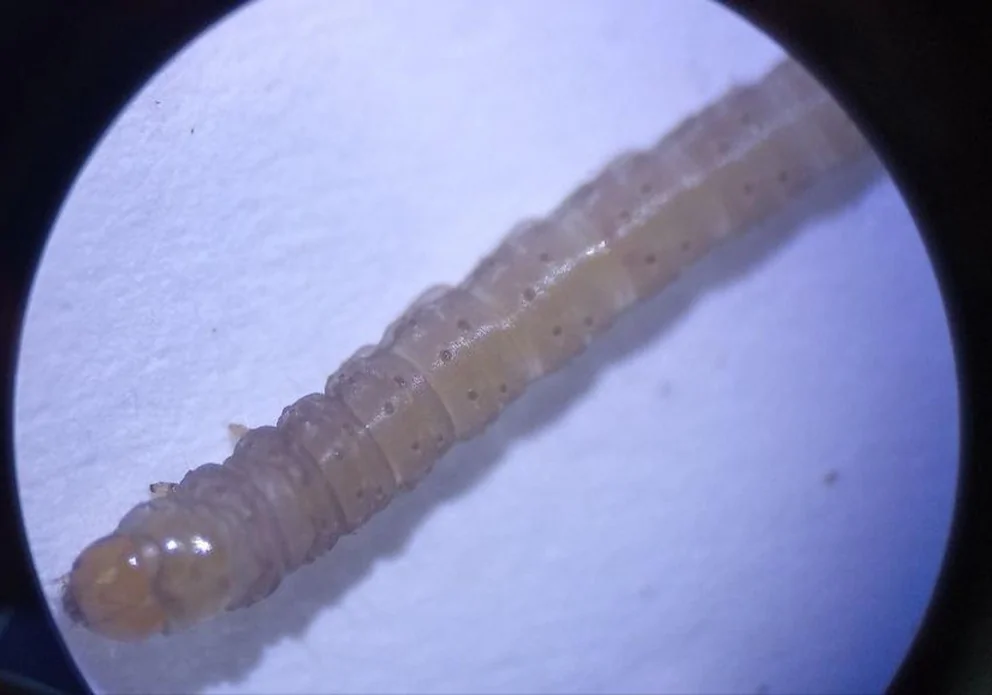
Cover image. Larva of a lepidopteran stem borer examined in this study, photographed under a dissecting microscope. Photo by the first author.

Figure 1 shows a larva, the focal organism investigated stemborers. The photo was taken under a dissecting microscope.

## 2 Materials and Methods

### 2.1 Study Site Description

**FIGURE 2:**
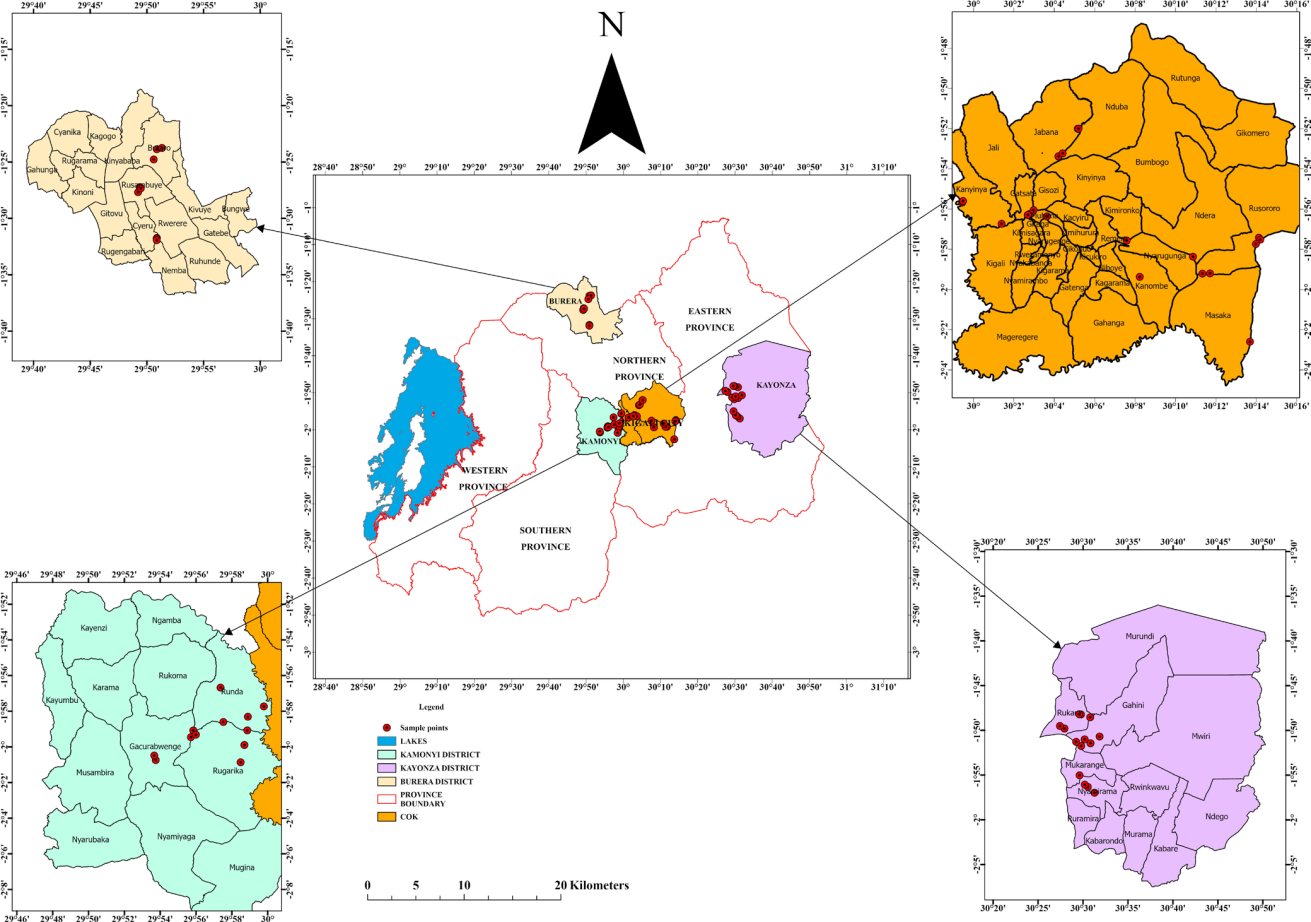
Geographical coverage of the study area with administrative boundaries that represent vegetation mosaics where the City of Kigali (COK) represents urban wetland & riparian mosaics (WR), Burera district represents Afro‐alpine mountain & Wetland mosaics (AMW), Kayonza district represents Akagera Subhumid Savanna and Wetland mosaics (ASW), and Kamonyi district represents Montane Forests and Wetland mosaics (MFW).

The study was carried out in four administrative regions representing the major vegetation mosaics and bioclimatic gradients of Rwanda, following the African phytogeographical classification (White 1983), East African vegetation mosaics (Lillesø et al. 2024), and the national classification of agro‐ecological zones of Rwanda (MINAGRI 2021; SANBI et al. 2022). Selected sites capture transitions between the Guineo–Congolese vegetation mosaic of the humid western highlands, the complex Sudanian woodland savanna of the eastern plains, and the Sudanian–Guineo–Congolese transitional zone (Figure 2).

In the eastern part of Rwanda, the Kayonza district, located at 1°45′–2°05′ S, 30°25′–30°50′ E, and 1200–1500 m altitude, was selected. It lies within the Sudanese regional mosaics, dominated by *Acacia‐Combretum* forests, bushland, and low‐altitude wetlands in a subhumid to semi‐arid climate (MINAGRI 2021; Lillesø et al. 2024; Ruticumugambi et al. 2024). In the northern part, the study was carried out in the Burera district, located at 1°21′–1°34′ S; 29°44′–29°54′ E, with an altitude > 2000 m in Afro‐alpine mountain and wetland mosaics (AMW), within the Guineo–Congolese regional center. This zone features montane grasslands dominated by *Pennisetum*, bamboo (*Yushania alpina*), and high‐altitude wetlands dominated by Typhaceae and Cyperaceae around Burera and Ruhondo lakes (SANBI et al. 2022; Nsengimana et al. 2025).

In the central part, Kigali City, located at 1°57′–1°59′ S; 30°03′–30°09′ E; 1300–1800 m above sea level, was selected as an urban wetland and river mosaic (WR) within the transition zone Sudanian–Guinean–Congo. Its landscape comprises urbanized hill–valley systems interspersed with Nyabarongo and Nyandungu wetlands, supporting riparian forests and secondary woodland–savanna mosaics with more modified greeneries (REMA 2012; ARCOS 2021; SANBI et al. 2022). In the southern part of the country, the Kamonyi district, located at 2°00′–2°15′ S, 29°45′–29°55′ E, and 1500–2000 m above sea level, dominated by Montane Forest and Wetland (MFM) mosaics, was selected. It is in a continuous transition zone with a large part lying within the Guineo–Congolese forest mosaic of the southern highlands. The mosaic structure in this zone is defined by canopy trees interspersed with herb‐rich wetlands, resulting in high biodiversity and a complex community (Mukuralinda et al. 2016; Iiyama et al. 2018; SANBI et al. 2022). Sampled localities within the vegetation mosaics were selected based on ecological composition, vegetation type, weather conditions, potential host plant availability, and accessibility (Table 1).

**TABLE 1:** Details on physico‐ecological characteristics and mosaic vegetation types of study areas (Lillesø et al. 2024; MINAGRI 2021; SANBI et al. 2022).

Province	District	Elevation (m)	Temperature (°C)	Rainfall (mm year^−1^)	Mosaics vegetation type
Northern	Burera	> 2000	19.4	1200–2200	Afroalpine mountain and wetland
Southern	Kamonyi	1500–2000	21.3	1000–1400	Montane forest and wetland
Eastern	Kayonza	< 1500	23.29	800–1000	Akagera sub‐humid savanna and wetland
Kigali City	Kigali City	1000–2000	21.9	900–1200	Wetland and riparian zones to the wetland

### 2.2 Study Design

Field surveys were conducted throughout the major climatic seasons of Rwanda in 2023–2024, including the long rainy season (March–May), the long dry season (June–September), the short rainy season (October–December), and the short dry season (January–March), to capture seasonal variation in stemborer occurrence and host plant use. The number of visits to every locality was at least three times, considering dry and long rainy seasons. At each locality, three to five people spent 2–6 h searching for infested plants depending on infestation levels. At each selected location, habitats harboring wild host plants from stemborers were systematically surveyed for stemborer infestations. These habitats included (i) the edges of the cultivated field and adjacent uncultivated zones, (ii) open patches along the forest access roads and pathways, (iii) riparian zones along urban areas, rivers, and streams, (iv) the edge of lakes, and (v) swamp ecosystems.

Within each habitat, plant species belonging to Poaceae, Cyperaceae, and Typhaceae were sampled. They were preferred based on previous records of their vulnerability to be infested by stemborers (Goftishu et al. 2018; Le Rü et al. 2006a, 2006b). Additionally, morphologically similar or closely related vegetation belonging to Cannaceae, Marantaceae, Juncaceae, and Zingiberaceae was also sampled. In particular, all plants that had leaf scarring, dead and desiccated shoots, frass deposits, and entry/exit holes were particularly targeted for stemborer sampling.

It is important to note that the sampling was not designed to be quantitatively standardized across host plant species. Instead, a purposive approach was employed, focusing primarily on plant individuals exhibiting visible signs of stemborer infestation like stems yellowing, leaf scarring, frass deposits, dead or desiccated shoots, and entry/exit holes. As a consequence, sampling effort varied among plant species depending on their availability, detectability, and infestation status in the field. Thus, plant species without observable symptoms of infestation were not studied, and therefore zero‐infestation observations are underrepresented in the dataset. This approach was adopted to maximize recovery of stemborer specimens in natural habitats but limited the ability to estimate absolute infestation rates across host plants and vegetation mosaics.

### 2.3 Data Collection

Each observed or suspected plant was carefully examined for visible signs of stemborer infestation. The infested plants were excised and dissected to recover the stemborer larvae and occasionally pupae, with eggs sometimes found on the surface of the plant. The recovered larvae were transferred to an artificial diet composed of maize flour, wheat germ, brewer's yeast, ascorbic acid, agar, and preservatives following standard protocols for lepidopteran stemborers prepared by ICIPE and partner experts. The diet was prepared under laboratory conditions and placed in plastic vials (7.5 × 2.5 cm), which were sealed with cotton wool (Overholt and Goebel 2001; Devi and Kaur 2015).

### 2.4 Identification of the Host Plant and the Stemborers

Host plants were identified in the field through direct observation of morphological characters and use of the iNaturalist platform (https://www.inaturalist.org/). When field identification was uncertain, photographs and representative specimens were examined and verified through consultation with plant taxonomists and botanists from the University of Rwanda, the Centre of Excellence in Biodiversity and Natural Resource Management (CoEB), Rwanda National Herbarium (NHR), and Rwanda Institute for Conservation Agriculture (RICA) experts to confirm the name of the plant species.

The identification of stemborers at the species level was performed using microscopic observations of morphological characteristics following the guide for stemborer identification keys developed by the International Center of Insect Physiology and Ecology (ICIPE) (Hailemichael et al. 2009), the recently published *Sesamia* species identification key (Le Rü et al. 2022), and the Lucid Central insects identification platform (https://www.lucidcentral.org). In addition, a stemborers taxonomic expert from ICIPE was consulted to validate the names of species. The unknown stemborer species were only identified at the genus level and are presented in their corresponding lowest taxonomic units, where the name is that of a genus based on differences in morphospecies. The voucher specimens are deposited in the zoology laboratory, Biology Department, College of Science and Technology, University of Rwanda, under the stemborers collection (access codes: KG, KM, KY, B 2023 & 2024). The letters represent the district or city where the specimens were collected, and the numbers indicate the year of collection.

### 2.5 Data Analysis

Abundance was calculated as the total number of stemborer individuals recovered from infested host plants. Because sampling was purposive and not standardized across host plant species or vegetation mosaics, abundance values represent raw counts rather than density estimates. Species diversity was assessed using the Shannon diversity (*H*′) and Simpson dominance (*D*) indices.

To investigate the structure of stemborer–host plant interactions, a bipartite ecological network was constructed using records of associations between each stemborer species and host plant species. The network was based on the presence and abundance of stemborer individuals recovered from each host plant and was used to evaluate patterns of host use and interaction structure among vegetation mosaics. Network analysis was conducted using the R packages bipartite, igraph, and network (R Core Team 2025). The following indices were calculated: network specialization (H2′), generality (HL), linkage density (Ld), and vulnerability (LL).

These indices describe the degree of interaction specialization within the network, the average number of host plants used by stemborers, the complexity of species interactions, and the average number of stemborer species associated with each host plant, respectively (Delmas et al. 2019; Almeida‐Neto and Ulrich 2011). These metrics were selected because they provide insights into the organization of plant–herbivore communities and are relatively robust to differences in network size and sampling intensity.

## 3 Results

### 3.1 Stemborers Relative Abundance and Diversity Among Ecological Mosaics

A total of 6098 stemborer individuals belonging to 18 species, 11 genera, and four families were identified, namely Crambidae, Noctuidae, Pyralidae, and Tortricidae (Table 3). Noctuidae was the dominant family with 11 species from five genera comprising *Acrapex*, *Busseola*, *Pirateolea*, *Poeonoma*, and *Sesamia*. The Crambidae family was represented by one genus and two species (*C*. *partellus* and *C. suppressalis*); Tortricidae was represented by two genera and two species (*Bactra* sp. and *Thaumatotibia* sp.), while Corossidae (*Carelis* sp.) and Pylaridae *(E. saccharina)* were represented by one genus and one species, respectively.

The highest number of species was recorded in urban wetlands and river mosaics, with 41.7% (*N* = 2536), and the Akagera subhumid savanna and its wetlands, with 27% (*N* = 1651) of the total specimens. The remainder was distributed in montane forests and their wetlands with 21% (*N* = 1282) and the Afro‐alpine mountains and their wetlands with 10.3% (*N* = 629) (Table 2). According to individual species preferences of ecological habitats, diversity indices showed that urban wetlands and riparian mosaics harbor the highest diversity (*H*′ = 2.02, *D*′ = 0.837), indicating a more even distribution of species, while Afroalpine mountain mosaics have the lowest diversity (*H*′ = 0.64, *D*′ = 0.263), suggesting a smaller number of species in the area compared to other surveyed areas.

**TABLE 2:** Abundance of stemborers and their distribution across ecological vegetation mosaics in Rwanda.

Stemborers				Abundance per mosaic type				
Family	No	Species	Author (s)	WR	ASW	AMW	MFW	Total
Noctuidae	1	*Acrapex* sp.	(Hampson, 1894)	6	0	0	3	9
	2	*Busseola fusca*	(Fuller, 1901)	649	975	538	711	2873
	3	*Busseola* sp.	(Thurau, 1904)	0	0	0	9	9
	4	*Pirateolea piscator*	(Fletcher, 1961)	61	7	0	5	73
	5	*Pirateolea* sp.	(Moyal et al. 2010)	0	3	0	0	3
	6	*Poeonoma serrata*	(Hampson, 1910)	44	2	5	15	66
	7	*Poeonoma* sp.	(Tams and Bowden, 1953)	0	0	2	0	2
	8	*Sesamia calamistis*	(Hampson, 1910)	168	37	0	47	252
	9	*Sesamia nonagrioides*	(Lefèbvre, 1827)	339	129	15	105	588
	10	*Sesamia* sp.	(Guenée, 1852)	17	0	0	6	23
	11	*Speia* sp.	(Stoll, 1790)	4	0	0	0	4
Crambidae	12	*Chilo partellus*	(Swinhoe, 1885)	567	282	31	194	1074
	13	*Chilo suppressalis*	(Walker, 1863)	148	91	0	41	280
Corossidae	14	*Carelis* sp.	(Bowden, 1956)	3	0	0	1	4
Pylaridae	15	*Eldana saccharina*	(Walker, 1865)	21	4	0	4	29
Tortricidae	16	*Bactra* sp.	(Stephens, 1834)	210	32	25	59	326
	17	*Thaumatotibia* sp	(Zacher, 1915)	299	89	13	82	483
			Total	2536	1651	629	1282	6098
			Relative abundance (%)	41.7	27	10.3	21	
			Species richness (S)	14	11	7	14	
			Shannon Index (*H*′)	2.02	1.35	0.64	1.54	
			Simpson Index (D)	0.837	0.609	0.263	0.654	

### 3.2 The Abundance and Distribution of Stemborer Species Among Wild Host Plants

Among economically important species, *B. fusca* , *C. partellus*, *S. calamistis*, and *S. nonagrioides* were the most dominant (Table 2). They sum up more than 78% of the total collected species. *B. fusca* itself counted 47.1% (*N* = 2873), followed by *C. partellus* with 17.6% (*N* = 1074) and *C. suppressalis* with 4.5% (*N* = 280). The *Thaumatotibia* genus was observed associated with small grasses and small *Cyperus* located in wetlands, with 7.8% (*N* = 483) compared to *Bactra* sp., with 5.3% (*N* = 326). In less economically important species, *Pirateolea* sp. was represented with 1.2% (*N* = 73), *Poeonoma* sp. with 1.08% (*N* = 68), and *E. saccharina* was less represented, with 0.47% (*N* = 29). Few species were rare in our collection and limited to very few individuals, where *Acrapex* sp. was nine, and *Carelis* sp. *and Speia* sp. were only four individuals each (Table 2).

Among the 56 identified wild host plants, the most infested species was *Pennisetum purpureum* Schumacher (family: Poaceae) with 29.53% (*N* = 1801), followed by *Carex cephalosphora* (family: Cyperaceae) with 10% (*N* = 611) and *Panicum* (family: Poaceae) with 5.3% (*N* = 327) stemborers. *Rottboellia cochinchinensis* and *Sorghum halepense* (L.) Pers. were less affected, with 3.7% (*N* = 228) and 3.6% (*N* = 225), respectively (Table 3).

**TABLE 3:** Stemborers species host preference and abundances per host plant.

No	Host plants		Number of stemborer by genera											Statistics			
	Species name	Niche	Ac	Bu	Ca	Ch	Ed	Se	Po	Pi	Bt	Th	Sp	Ng	Ia	*H*′	*D*
	Poaceae																
1	*Arundo donax* L.	*G*	1	0	0	1	0	0	0	0	0	0	0	2	15	0.39	0.23
2	*Chrysopogon zizanioide s (L.) Roberty*	*G*	0	1	0	1	0	1	0	0	0	0	0	3	78	0.95	0.56
3	*Coix lacryma‐jobi*	*G*	0	0	0	1	0	0	0	0	0	0	0	1	8	0.00	0.00
4	*Cenchrus purpureus*	*G*	0	0	0	1	0	1	0	1	0	0	0	3	39	0.81	0.47
5	*Cymbopogon citratus*	*G*	0	0	0	1	0	0	1	0	1	0	0	3	11	0.86	0.51
6	*Echinochloa crus‐galli*	*G*	0	0	0	2	0	2	0	0	0	0	0	4	32	0.51	0.27
7	*Echinochloa frumentacea*	*G*	0	1	0	1	0	2	0	0	0	0	0	4	67	1.57	0.75
8	*Echinochloa pyaramidalis*	*S, Aq*	0	1	0	2	0	3	1	1	0	0	0	8	119	1.28	0.62
9	*Eleusine coracana*	*S, Dl*	0	0	0	2	0	0	0	0	1	1	0	4	64	1.62	0.76
10	*Hyparrhenia hirta*	*Dl*	0	0	0	1	0	0	0	1	1	1	0	4	23	1.30	0.66
11	*Hyparrhenia papillides* (Hochstetter) Stapf	*Dl*	0	0	0	1	0	0	0	0	0	1	0	2	38	1.02	0.62
12	*Hyperthelia dissoluta* (Steudel) Clayton	*Dl*	0	0	0	1	0	0	0	0	1	0	0	2	41	0.77	0.50
13	*Imperata cylindrica*	*Ae*	1	0	0	2	0	0	0	0	0	0	0	3	42	0.79	0.45
14	*Lolium* sp.	*G*	0	0	0	1	0	0	0	1	2	0	0	4	23	0.84	0.50
15	*Miscanthus Variegatus*	*Ae*	0	0	0	1	0	0	0	0	0	0	0	1	7	0.00	0.00
16	*Panicum maximum*	*H*	0	1	1	2	0	2	0	1	0	0	0	7	245	1.36	0.64
17	*Panicum ripens*	*H*	0	0	0	1	0	2	1	0	0	0	0	4	82	1.32	0.71
18	*Paspalum* sp.	*G*	0	0	0	2	0	0	0	0	2	0	0	4	99	0.89	0.52
19	*Pennisetum clandestinum*	*G*	0	1	0	1	0	0	0	0	0	0	0	2	86	0.89	0.52
20	*Pennisetum macrourum*	*G*	0	0	0	1	0	1	1	1	0	0	0	4	34	1.02	0.55
21	*Pennisetum purpureum* Schumacher	*G*	0	1	0	1	0	1	2	0	0	0	0	4	1801	0.31	0.17
22	*Pennisetum polystachion*	*G*	0	1	0	1	0	0	0	0	0	0	0	2	87	0.05	0.01
23	*Pennisetum thunbergii*	*G*	0	1	0	1	0	1	1	0	0	0	0	4	63	1.36	0.68
24	*Phragmites* sp.	*H, Ae*	0	1	0	1	1	2	0	1	0	0	0	6	199	1.48	0.73
25	*Rottboellia cochinchinensis* (Loureiro) Clayton	*G*	0	1	0	1	0	2	0	0	0	0	0	4	228	1.02	0.62
26	*Rottboellia Exaltata*	*H*	0	0	0	0	0	0	0	0	1	0	0	1	16	1.08	0.54
27	*Saccharum officinarum* L.	*G*	0	0	0	0	13	0	0	0	0	0	0	1	13	0.00	0.00
28	*Setaria megaphylla* (Steudel) T. Durand. & Schinz	*H*	0	0	0	1	0	0	0	0	2	0	0	3	44	0.69	0.50
29	*Setaria sphacelata*	*H*	0	0	0	1	0	0	0	0	1	0	0	2	67	0.63	0.44
30	*Sorghum arundinaceum* (Desvaux) Stapf	*G*	0	1	0	1	0	1	1	1	0	0	0	5	46	1.36	0.66
31	*Sorghum bicolor* (L.) Moench	*G*	0	1	0	1	0	0	0	0	0	0	0	2	127	0.30	0.16
32	*Sorghum halepense* (L.) Pers.	*G*	0	1	0	1	0	0	1	0	0	0	0	3	225	0.57	0.30
33	*Tripsacum* sp.	*G*	0	1	0	0	0	0	0	0	0	0	0	1	75	0.18	0.08
34	*Zizania latifolia*	*Aq*	0	0	0	2	0	0	1	0	2	0	0	5	142	0.44	0.22
35	*Zea mays* L.	*G*	0	1	0	1	0	1	0	0	0	0	0	3	176	1.16	0.64
	Cyperaceae																
36	*Carex cephalosphora*	*H*	0	0	0	0	0	1	0	0	1	0	0	2	611	0.69	0.50
37	*Carex chlorosaccus* C.B. Clarke	*H*	0	0	0	0	0	1	0	0	1	0	0	2	68	0.78	0.44
38	*Cyperus articulatus* L.	*H, Aq*	0	0	0	0	0	1	0	0	1	1	0	3	142	1.04	0.59
39	*Cyperus difformis*	*H, Aq*	0	0	0	0	0	2	0	0	1	1	0	4	162	0.94	0.56
40	*Cyperus distans* L.	*H, Aq*	0	0	0	1	0	1	0	1	0	1	0	4	126	1.16	0.65
41	*Cyperus dives* Del.	*H, Aq*	0	0	0	0	0	1	0	0	0	1	0	2	112	0.66	0.47
42	*Cyperus exaltatus* Retzius	*H, Aq*	0	0	0	0	0	1	0	1	0	1	0	3	88	0.91	0.55
43	*Cyperus latifolius*	*H, Aq*	0	0	0	0	0	1	0	1	0	1	0	3	37	1.06	0.64
44	*Cyperus odoratus*	*H, Aq*	0	0	0	0	0	0	0	0	0	1	0	1	27	0.00	0.00
45	*Cyperus prolifer Lamark*	*H, Aq*	0	0	0	0	0	0	0	0	0	1	0	1	6	0.00	0.00
46	*Cyperus strigosus*	*H, Aq*	0	0	0	0	0	1	0	1	0	1	0	3	21	0.91	0.56
47	*Schoenoplectus*	*H, Aq, Il*	0	0	0	1	0	1	0	0	0	1	0	3	72	1.01	0.61
48	*Scleria gaertneri Raddi*	*H, Aq*	0	0	0	0	0	1	0	0	0	1	0	2	15	0.64	0.44
	Typhaceae																
50	*Typha domingensis*	*Aq, R*	0	0	0	0	0	1	0	0	0	1	1	2	32	0.60	0.32
51	*Typha latifolia*	*Aq, R*	0	0	0	0	0	1	0	1	0	1	0	3	79	1.20	0.61
	Juncaceae																
52	*Juncus effusus* (New)	*H, Aq, Il*	0	0	0	1	0	1	0	0	1	0	0	3	37	1.32	0.72
	Marantaceae																
53	*Thalia dealbata* (New)	*Ae*	0	1	0	0	0	0	0	0	0	0	0	1	5	0.00	0.00
	Cannaceae																
54	*Canna indica* (New)	*Ae*	0	0	0	0	0	1	0	0	0	0	0	1	6	0.00	0.00
	Zingiberaceae																
55	*Etlingera* sp. (New)	*Ae*	0	1	0	0	0	0	0	0	0	0	0	1	7	0.00	0.00
56	*Alpinia* sp. (New)	*Ae*	0	0	0	0	1	0	0	0	0	0	0	1	3	0.00	0.00

Abbreviations: Ac, *Acrapex*; Bt, *Bactra*; Bu, *Busseola*; Ca, *Carelis*; Ch, *Chilo*; D, Simpson index; Ed., *Eldana*; *H*′, Shannon index; Ia, Individual abundance of stemborers per host plant; Ng, number of genera per host plant; Pi, *Pirateolea*; Po, *Poeonoma*; Se, *Sesamia*; Sp: *Speia*; Th, *Thaumatotibia*.

### 3.3 Host Plant‐Stemborer Interaction Network

**FIGURE 3:**
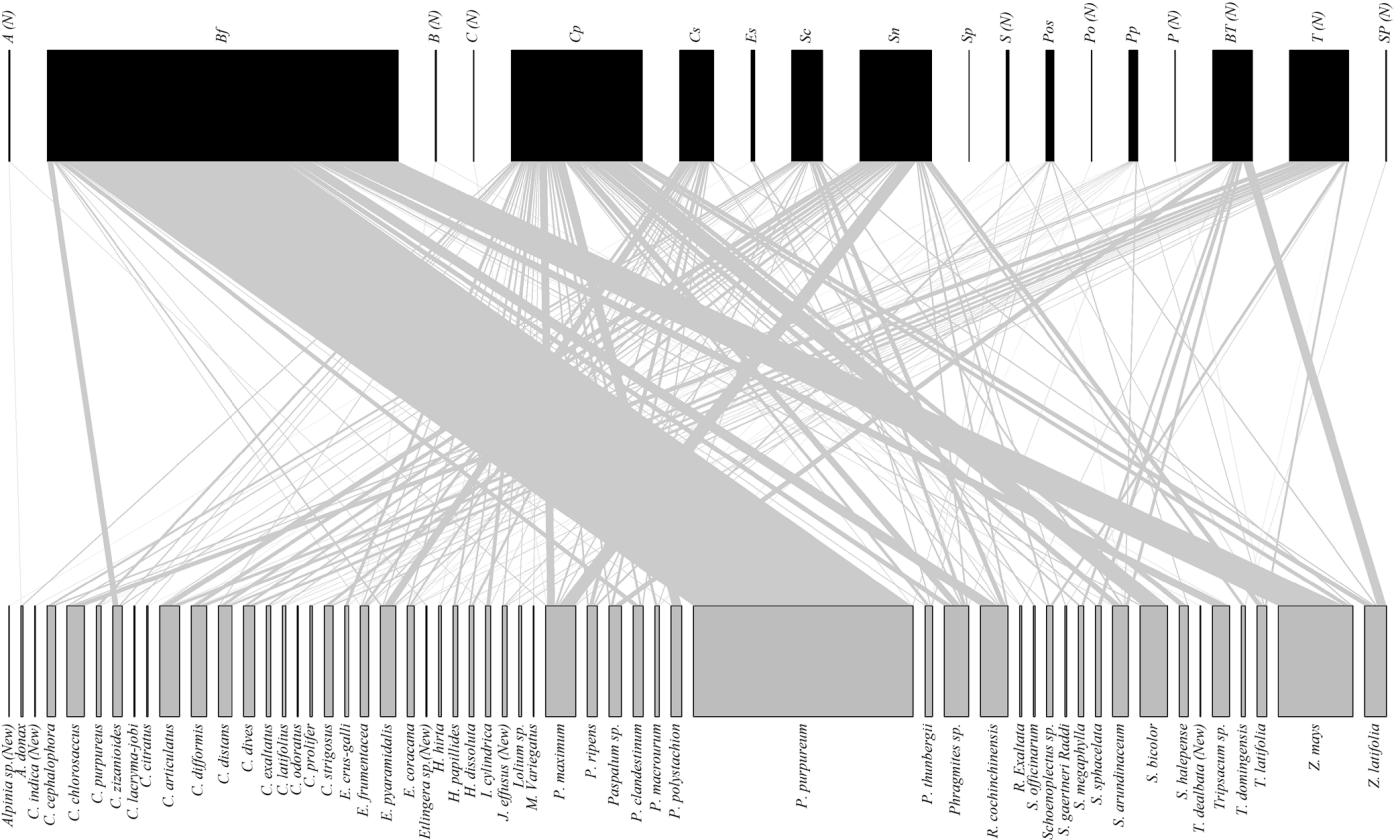
Stemborer–host plant interaction network of complete data set showing connections between stemborer species (top) and host plants (bottom). Each top and bottom rectangle represents the proportional abundance of stemborers and host plants, and each link thickness indicates interaction strength. The abbreviations of the stemborer species refer to Table 2, while plants refer to Table 3.

A complete dataset of the analysis of the plant‐stemborer interaction network comprising 56 plant species and 18 stemborer species specifically shows that 64.2% (*N* = 36) of the host plant species belong to Poaceae, while 23.6% (*N* = 13) of the plant species belong to Cyperaceae. The host plant families Typhaceae and Zingiberaceae were represented by two individuals only for each family. The remaining families were represented by a single species (Table 3). The analysis of network indices showed a high linkage density (Ld = 5.86), indicating multiple associations between species at trophic levels. The specialization index (*H*′_2_ = 0.63) reflects moderate niche partitioning, while the analysis of stemborer species connections showed high generality (HL = 9.57). Furthermore, host plants showed moderate vulnerability (LL = 2.15) to being attacked by an average of two stemborer species (Figure 3).

The analysis of subdataset on host plant distribution in surveyed areas shows that urban wetlands and their riparian mosaics accounted for 83.9% (*N* = 47), followed by montane forest and its wetlands, with 58.9% (*N* = 33) of the total host plants recorded. The subhumid savanna of Akagera and its wetlands represented 55.3% (*N* = 31), while the Afro‐alpine mountains and their wetland mosaics had the lowest abundance of host plants, with 12.5% (*N* = 7). In addition, five host plants of stemborers, namely *Canna indica* (Cannaceae), *Thalia dealbata* (Marantaceae), *Juncus effusus* (Juncaceae), and two species of *Etlingera* sp. and *Alpinia* sp. (Zingiberaceae), were observed hosting lepidopteran stemborers for the first time. Besides, rare stemborer host plants known as *Schoenoplectus* and *Scleria gaertneri Raddi* from the Cyperaceae family are listed in the study.

**FIGURE 4:**
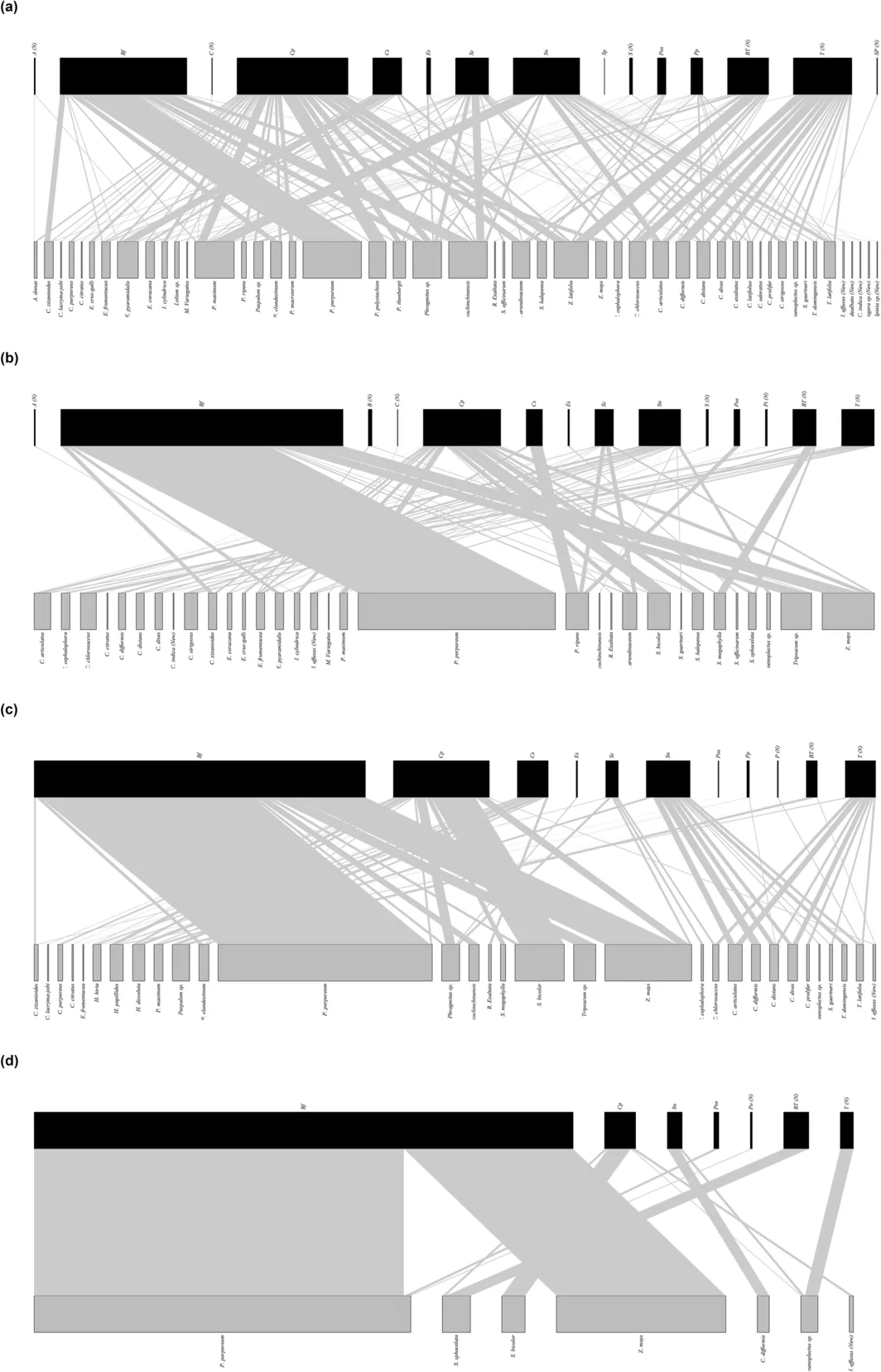
Semborer–host plant interaction networks across four ecological biomes of Rwanda. (a) Urban Wetlands and Riparians (WR), (b) Akagera Sub‐Humid Savanna and Wetland (ASW), (c) Montane Forest and Wetland (MFW), and (d) Afroalpine Mountain and Wetland (AWM). The top is stemborer species, the bottom is the host plant, black bars represent stemborer species, and gray bars represent host plant species, with bar widths proportional to species abundance. Connecting lines indicate stemborer–host plant interactions, and line thickness reflects interaction frequency.

The analysis of the ecological interaction network of stemborer‐host plants in four ecological vegetation mosaics shows a different structure of the interaction network (Figure 4). Urban wetlands and their riparian mosaics (Figure 4a) showed the highest density of linkage (Ld = 6.38) and generality (HL = 10.15), along with the lowest specialization (*H*′_2_ = 0.58). In contrast, the Afro‐alpine mountain and its wetlands (Figure 4d) exhibited the lowest link density (Ld = 1.46) and generality (HL = 1.82), but the highest specialization (*H*′_2_ = 0.95). Intermediate values were observed for the montane forest and its vegetation mosaic of wetlands (Figure 4d) (Ld = 3.15; *H*′_2_ = 0.75; HL = 4.69) and the subhumid savanna of Akagera and its wetlands (Figure 4c) (Ld = 3.00; *H*'_2_ = 0.74; HL = 4.48). The vulnerability analysis of the ecological mosaics to stemborers showed a similar trend, where urban wetlands and riparian zones were the most exposed (LL = 2.60) while the Afroalpine mountain was the least exposed to stemborer attacks (LL = 1.11) (Figure 4).

## 4 Discussion

This study provides the first comprehensive overview of lepidopteran stemborer diversity and their associated wild host plants across major ecological vegetation mosaics in Rwanda. A total of 18 stemborer species were recorded from 56 host plant species, revealing a diverse and previously under‐documented community of herbivores in natural and semi‐natural habitats. The coverage of multiple ecological regions, including southern, northern, eastern, central, and urban modified habitats, allowed for a broad characterization of species occurrence and host associations across distinct vegetation mosaics. However, given the ecological heterogeneity of Rwanda and the exclusion of the western province, which is recognized as one of the country's most biodiversity‐rich regions (ARCOS 2021; SANBI et al. 2022; Lillesø et al. 2024), it is likely that additional species and host associations remain undocumented. Expanding surveys to include all ecological zones would provide a more complete understanding of stemborer population, as well as potential variation in species composition and host use across habitats.

In wild habitats, lepidopteran stemborers are found mainly in thick grasses and sedges that grow in wetter localities (Le Rü et al. 2006b; Assefa et al. 2010; Moyal 2014; Goftishu et al. 2018). In this study, urban wetlands and their associated riparian zones appeared to support relatively high stemborer diversity and abundance compared to other surveyed areas. Although this pattern should be interpreted cautiously due to the purposive and non‐standardized sampling design, it nonetheless highlights an important ecological signal. These habitats are rich in small and medium wetlands, riparian vegetation, gardens, and small‐scale green spaces that are characterized by high structural heterogeneity and a wide diversity of potential host plants (Lillesø et al. 2024; SANBI et al. 2022), which can collectively support complex herbivore communities such as stemborers (Overholt and Goebel 2001; Le Rü et al. 2006b; Moolman et al. 2014; Goftishu et al. 2018).

Within human‐dominated landscapes such as Kigali City, these urban and peri‐urban environments may negatively affect species community structure, or create microhabitats that maintain species interactions despite ongoing anthropogenic pressures. This may explain how ongoing urban expansion and fragmentation of natural habitats may alter the ecological roles of certain stemborer species. Species that were previously confined to natural ecosystems and not considered economically significant may progressively adapt to modified habitats and emerge as potential pests in ornamental gardens and small‐scale urban farming systems. Initially, most economically important stemborers co‐existed with farming fields and were not playing any economic importance (Khan et al. 1997, 2007; Assefa et al. 2015).

The hypothesis of shifting is supported by host switching behavior in stemborers, particularly among closely related plant species (Calatayud et al. 2014; Jones et al. 2022; Ntirenganya et al. 2026), and stemborers tend to perform better on domesticated crops than on their wild ancestors due to reduced levels of secondary metabolites during crop domestication (Fernandez et al. 2021; Badenes‐Pérez 2025). The resource concentration hypothesis further explains that monocultures or high‐density stands of a single crop species facilitate the specialization and population buildup of herbivorous insects (Otway et al. 2005; Deng et al. 2022). Indigenous African lepidopteran stemborers are believed to have coevolved with native grasses and sedges and subsequently expanded their host range to cultivated cereals following anthropogenic changes in land use (Polaszek and Khan 1998; Sezonlin et al. 2006). Historical evidence of the colonization of *E. saccharina* from wild grasses to sugarcane in South Africa and Zimbabwe supports the hypothesis (Conlong 2001; Sezonlin et al. 2006; Le Rü et al. 2006b; Moolman et al. 2014; Goftishu et al. 2018). In this context, urban regreening and eco‐park development initiatives offer important opportunities to balance biodiversity conservation and ecosystem function. Prioritizing native vegetation in these interventions may help to restore more natural plant–insect interaction networks, support the persistence of insect communities within their original ecological niches, and potentially reduce the risk of pest emergence in highly modified urban environments (Morpurgo et al. 2024; Bowler et al. 2025).

Similarly to our findings, a high diversity of noctuid stemborers was reported in humid and grassland mosaics in the East (Goftishu et al. 2018; Ong’amo et al. 2018), South (Moolman et al. 2014), and West Africa. And the observed lower occurrence of rare stemborer species was reported in similar ecosystems. These studies reported rare or less common species in natural habitats in eastern (Goftishu et al. 2018; Le Rü et al. 2006b, 2006a), southern (Moeng et al. 2018; Moolman et al. 2014), and western (Ndemah et al. 2007; Hailemichael et al. 2009; Ong'amo et al. 2014) regions of Africa. Most of these less acounted species were *Acrapex, Bactra, Carelis, Pirateolea* (Goftishu et al. 2018; Le Rü et al. 2014; Moyal et al. 2010; Ong’amo et al. 2018), and *Speia* genera (Moolman et al. 2014), which is the same to this study's findings. Unlike a previous study that reported *Poeonoma* species distribution from *P. purpureum* in Rwanda (Le Rü et al. 2020), this study added their association to wetter ecosystem grasses. However, the presence of these less accounted species may not necessarily indicate sampling artifacts, since high sampling efforts increase the incidence of rare species (Le Rü et al. 2014).

Our study documented the response of key economically important stemborer species to altitudinal gradients. Beyond its distribution, *B. fusca* was the only species that colonized the high altitude of the Afro‐alpine mountain zone of the northern part of Rwanda and was present in higher numbers in other zones. In addition, *C. partellus* dominated the low altitude of the Akagera subhumid savanna in the eastern part of Rwanda and coexisted with *Sesamia* species at the mid‐altitude transition zones between the low altitude of the Akagera subhumid savanna and the high altitude of the Afroalpine mountain zones. Some studies reported that *B. fusca* dominated high altitudinal zones, while *C. partellus* dominated low altitudinal zones (Mutamiswa et al. 2022; Mwalusepo et al. 2018).

The findings alert the migration and shifting of both species to new ecological zones, as alarmed by Getu et al. (2001). Previous studies conducted on crops indicated that these species are potential threats to East African maize and other cereal crop systems, particularly in Rwanda (Ntirenganya et al. 2025, 2026), Ethiopia (Getu et al. 2001; Tefera 2004; Assefa 2006; Melaku et al. 2006; Tamiru et al. 2007; Assefa et al. 2010; Asmare et al. 2014; Goftishu et al. 2017), Kenya (Kanya et al. 2004; Ong'amo 2005, 2009; Otieno et al. 2008), and South Africa (Overholt and Goebel 2001; Moolman et al. 2014; Zhou 2016; Assefa et al. 2017). In addition, these studies reported species such as *S. nonagrioides, S. calamidtis*, and *E. saccharina* as wild habitat species that may appear in crops without causing critical harm. However, this ecological dynamic of shifting in species' natural habitats underscores the potential risk of “new” pest emergence from species currently confined to natural habitats or new pest outbreaks in new ecological zones that could be a threat to cereal crop systems and may increase the investments in pest management.

Our study observed an unusual pattern, with a remarkably high abundance of *Thaumatotibia* individuals infesting sedges (*Carex* and *Cyperus*, family Cyperaceae) and a smaller presence in grasses (*Eleusine*, *Hyparrhenia*, and *Hyperthelia*, family Poaceae). This is particularly striking given that most of the previously known members of *Thaumatotibia* are globally recognized as fruit borers, primarily infesting citrus, stone fruits, and macadamia nuts (European Food Safety Authority (EFSA) et al. 2020). To date, there have been no documented reports of species belonging to the genus *Thaumatotibia* that behave as stemborers and feed on plant stems. This unexpected finding alarms an important ecological, taxonomic, or host expansion question and behavioral shift or the presence of an undescribed or misclassified species within the genus. Given the importance of quarantine and the polyphagous nature of *Thaumatotibia* species, such as *T. leucotreta* (false codling moth) (European Food Safety Authority (EFSA) et al. 2020), this observation interpretation remains speculative without molecular data and further evidence to confirm the species and feeding behavioral changes.

The borer–host plant network analysis revealed that most stemborer species exhibited a narrow host range, indicating a high degree of host specialization. However, some species were associated with a broader range of host plants. The oligophagous species *B. fusca* and *C. partellus* utilized a greater number of host plants, although these hosts largely belonged to the same or closely related plant families, suggesting phylogenetic constraints in host selection. In contrast, several other borer species displayed a monophagous feeding strategy, being restricted to one or very few host plants, often within the same genus or family. These findings highlight varying degrees of host specificity among stemborers, ranging from strict specialization to limited generalization.

No polyphagous stemborer species were observed utilizing host plants from distantly related plant families, indicating a generally high degree of host specialization. Similar patterns have been reported in stemborer‐host plant utilization in Ethiopia and Tanzania (Goftishu et al. 2018; Ong’amo et al. 2018) as well as in other groups of insects (Wyckhuys et al. 2024; Bassi and Staude 2024). From an ecological network perspective, such specialization suggests that the loss of host plant species could alter network structure and connectivity, potentially leading to declines or local extinction of associated stemborer species due to their strong dependence on a limited number of hosts. The loss of a species may also disrupt broader ecological interaction networks and the functions they support, including trophic relationships involving natural enemies and other associated ecosystem services (Nyman et al. 2007; Neff et al. 2021; Wang et al. 2023).

In natural and normal conditions, the high index of linkage density and generality explains higher community stability (Batáry et al. 2021), while the low indices indicate lower trophic levels (Wang et al. 2023). This study reports comparatively higher indices in fragmented urban wetlands and their riparian zones than in the remaining vegetation mosaics. These effects may be associated with anthropogenic activities, particularly the domestication and cultivation of small‐scale, nonnative wild plant species for horticultural and esthetic purposes in household gardens located in urban and peri‐urban landscapes (Morpurgo et al. 2024; Bowler et al. 2025), which are morphologically or physiologically similar to stemborer hosts. Such urban‐driven vegetation structures can create microhabitats that may reshape vegetation structure (Alghali 1985; Moolman et al. 2014; Deng et al. 2022) and attract or assist the survival of arthropods like stemborer or serve as their refuge (Morpurgo et al. 2024; Bowler et al. 2025).

Furthermore, with the latter discussed factor, we observed new stemborer‐host associations with plants belonging to Cannaceae, Marantaceae, Juncaceae, and Zingiberaceae, previously unreported. Such observations may reflect the capacity of stemborers to exploit novel resources in fragmented landscapes such as rapidly urbanizing areas, as previously reported in urban and intensified agricultural landscapes (Planchuelo et al. 2020; Tartaglia and Aronson 2024). However, although this represents a noteworthy finding of the present study, these observations are based on the presence of larvae within plant tissues and do not alone confirm that these plants serve as true developmental hosts. Accordingly, these newly recorded plant species and families should be considered as potential or putative hosts rather than confirmed host plants. These records provide important preliminary evidence of potential host use; however, confirmation of their host status will require further investigation. Future studies should combine experimental validation through full life‐cycle rearing and controlled host suitability tests with molecular characterization of both stemborer specimens and their feeds to validate species identities and associated host plants, to better understand the ecological and evolutionary significance of these interactions in faster‐changing landscapes (Planchuelo et al. 2020; Tartaglia and Aronson 2024).

## 5 Conclusions

This study documented 18 lepidopteran stemborer species associated with 56 host plant species across major agroecological vegetation mosaics of Rwanda. The widespread occurrence of *Busseola fusca* (Fuller) (Noctuidae) and *Chilo partellus* (Swinhoe) (Crambidae), the two most economically important stemborers, highlights their broad ecological distribution and alarms ongoing expansion beyond their traditionally recognized ecological niches. Although urban wetlands and riparian mosaics appeared to support greater stemborer and host plant diversity than other surveyed habitats, comparisons among vegetation mosaics should be interpreted cautiously because sampling was purposive and not standardized across habitats and host plant species.

While most species were associated with wild grasses and sedges, several were also recorded on fodder grasses widely adopted by farmers on farm boundaries or around for soil stabilization, livestock feed, or other domestic use. This finding raises concerns about the potential exchange of stemborer pests between wild and cultivated systems and reinforces recommendations that fodder grasses be maintained at appropriate distances from cereal crops, consistent with established push‐pull management practices.

In addition, the fruit‐feeding genus *Thaumatotibia* was unexpectedly observed feeding within the soft stems of wetland vegetation, and potential new stemborer associations were recorded with representatives of the Cannaceae ( *Canna indica* ), Marantaceae ( *Thalia dealbata* ), Juncaceae ( *Juncus effusus* ), and Zingiberaceae (*Etlingera* sp. and *Alpinia* sp.) families. However, these observations should be regarded as preliminary evidence of possible novel host use rather than confirmed cases of host expansion. Because several of these insect‐host associations are reported for the first time, future studies should combine molecular characterization of stemborers and feeding behaviors to validate host plant associations, strengthen host records, and clarify the ecological and evolutionary significance of these interactions. In addition, controlled rearing experiments and life‐cycle completion studies are needed to confirm host suitability and improve understanding of stemborer ecology across East African ecosystems.

## Author Contributions

**Elie Ntirenganya:** conceptualization (equal), formal analysis (equal), funding acquisition (equal), investigation (equal), methodology (equal), writing – original draft (equal), writing – review and editing (equal). **Muluken Goftishu:** conceptualization (equal), methodology (equal), supervision (equal), validation (equal), visualization (equal), writing – review and editing (equal). **Yoseph Assefa:** supervision (equal), validation (equal), writing – review and editing (equal). **Bonoukpoè Mawuko Sokame:** methodology (equal), supervision (equal), writing – review and editing (equal). **Simon Boniface Boni:** supervision (equal), writing – review and editing (equal). **Janvier Uwayezu:** formal analysis (equal), software (equal), writing – review and editing (equal). **Hategekimana Athanase:** formal analysis (equal), software (equal), supervision (equal), visualization (equal), writing – review and editing (equal). **Venuste Nsengimana:** conceptualization (equal), data curation (equal), methodology (equal), supervision (equal), validation (equal), writing – review and editing (equal).

## Funding

This work was funded by the Partnership for Applied Skills in Sciences, Engineering, and Technology–Regional Scholarship and Innovation Fund (PASET–RSIF) and Carnegie Corporation of New York.

## Conflicts of Interest

The authors declare no conflicts of interest.

## Supporting information


**Data S1:** Stemborers–host plants supporting data.

## Data Availability

The data that supports the findings of this study are available in the Supporting Information of this article.
